# Distinguishing fanged frogs (*Limnonectes)* species (Amphibia: Anura: Dicroglossidae), from Thailand using high resolution melting analysis

**DOI:** 10.1038/s41598-023-43637-2

**Published:** 2023-10-30

**Authors:** Chatmongkon Suwannapoom, Maslin Osathanunkul

**Affiliations:** 1 School of Agriculture and Natural Resources, University of Phayao, Muang District, Phayao, Thailand; 2 Department of Biology, Faculty of Science, Chiang Mai University, Muang District, Chiang Mai, 50200 Thailand

**Keywords:** Molecular biology, Biological techniques

## Abstract

Morphologically, species of fanged frogs (*Limnonectes*) are exceedingly similar, making it difficult to distinguish them within the complex. In Thailand, it has been difficult to distinguish between the sympatric species *L. bannaensis* and *L. taylori*, particularly among tadpoles, adolescents, and adult females. A precise identification contributes to a greater understanding of biodiversity, particularly for assessing distributions and population dynamics. Therefore, a novel approach is required. The objective of this study was to develop a high resolution melting analysis (HRM) for the rapid and accurate identification of six species of *Limnonectes* of the *L. kuhlii* complex found in Thailand, particularly the two sympatric fanged frogs. Here, HRM assays using 16S rRNA mitochondrial primers were designed and developed. There was as much as a 25.3% variation in the nucleotide sequence of the fragment amplified by HRM16S primers among the six species of *Limnonectes*. Prior to conducting an in vitro HRM, the DNA sequences were used in a simulation HRM, uMELT Quartz, to predict the melting curve for each species of *Limnonectes*. There were discrepancies between the predicted melting curves of each species generated by the programme. Consequently, in vitro HRM tests were conducted. The obtained melting curve and T_m_ values were consistent with those predicted, albeit with a slightly different T_m_ value and a more distinct melting curve. All evaluated species of *Limnonectes* could be easily distinguished from one another by comparing the melting curve shapes. The HRM assay was then used to confirm the species of 18 *Limnonectes* samples in comparison to the reference samples (confidence interval > 90%). In addition, the results of HRM were consistent with those of experts who used morphological analysis to identify species. The HRM was found to be useful, and therefore the method would also contribute to future ecological and systematic studies on the target species.

## Introduction

**Figure 1:**
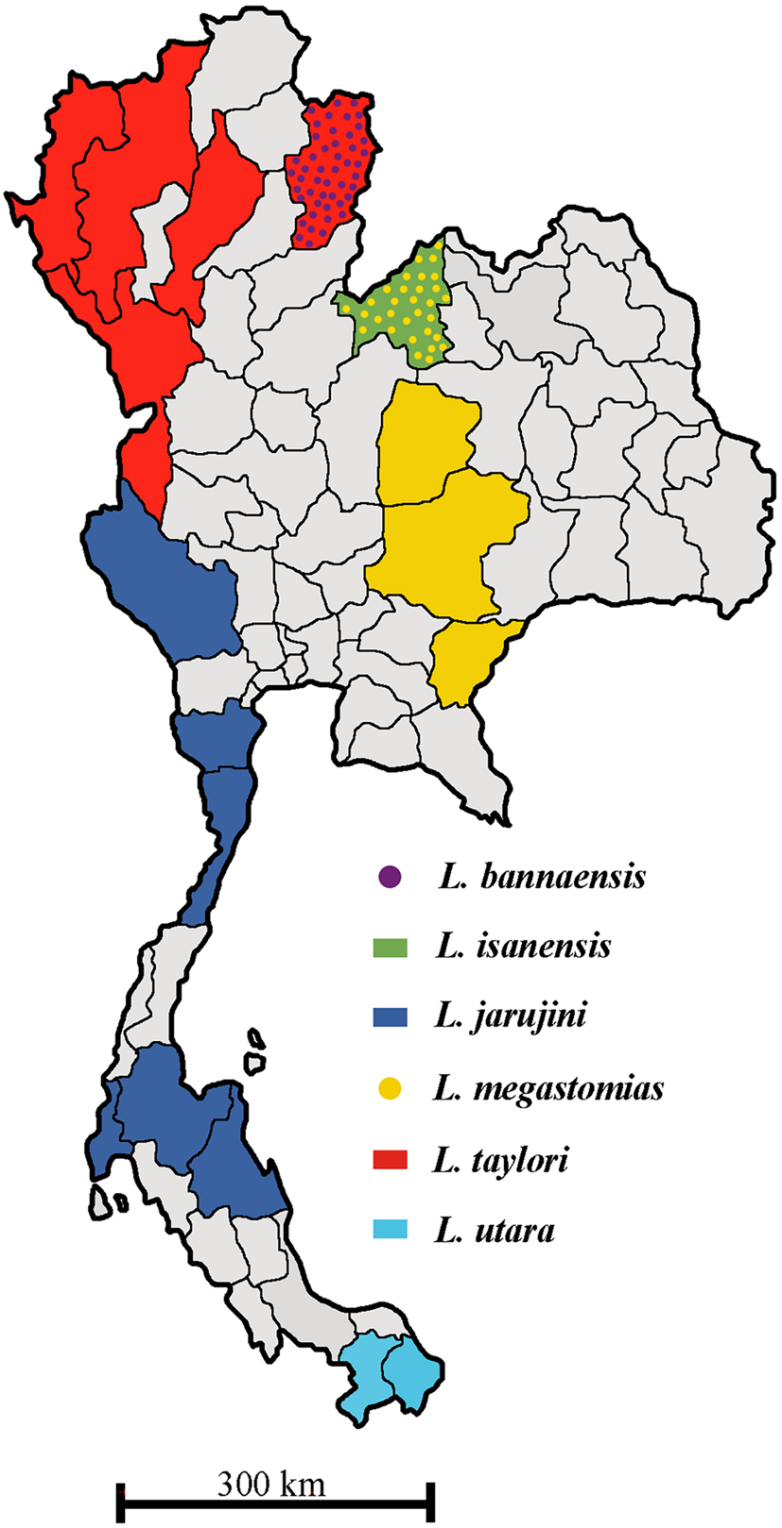
Distribution of six currently recognised *Limnonectes* species in Thailand.

*Limnonectes* Fitzinger presently contains 74 species throughout east and southeast Asia (Frost 2020). *Limnonectes* are potentially important for biodiversity assessments, but there now appear to be many more cryptic species than previously estimated. It is speculated that cryptic species were predominantly found in reptiles and insects and were more likely to be found in tropical rather than temperate regions^1^. *Limnonectes* likely contains numerous undescribed cryptic species, especially in widespread complexes such as the *L. kuhlii* complex^2^. Across its range, *Limnonectes* displays an extraordinarily high level of morphological similarity. The six currently recognised *Limnonectes* species are distributed throughout Thailand^3^, ranging from the north to the south of the country (Fig. 1). *L. taylori* ranges from the northwestern region of Thailand (Chiang Mai, Mae Hong Son, Lam Pang, Nan, and Tak Provinces); *L. jarujini* ranges from southwestern to southern (peninsular) Thailand (Kanchanaburi, Uthai Thani, Petchaburi, Prachuap Kirikhan, Phang Nga, Surat Thani, and Nakhon Si Thammarat Provinces); *L. bannaensis* ranges known only from Nan province; *L. megastomias* known only from east-central Thailand in Loei, Chaiyaphum, Nakhon Ratchasima, and Sa Kaeo provinces; *L. isanensis* known only from Loei Province; and *L. utara* from peninsular Thailand (Narathiwat Province). Interestingly, Matsui et al.^4^ once thought that *L. taylori* was the only species in the *L. kuhlii* complex found in the north and *L. jarujini* was inhabited in the south of Thailand. Recently, the occurrence of *L. bannaensis* and *L. taylori* was described from the same location, Bo Kluea, Nan Province, in the northern part of Thailand^5^. Morphologically, these two species are extremely close, and it has been difficult to distinguish their tadpoles, juveniles, and adult females^5^. Accurate and rapid species identification is crucial for assessing distributions and population dynamics, so a new approach is needed to better distinguish them.

Recently, several studies reported applying high resolution melting (HRM) in species identification or authentication. These studies showed that HRM could effectively distinguish plants (e.g., Refs.^6–8^) animals (e.g., Refs.^9–11^), and bacteria (e.g., Refs.^12,13^). Basically, HRM analysis characterises nucleic acid samples based on their disassociation behaviour and detects sequence differences in PCR-amplified sequences. Samples are discriminated based on their composition, length, and guanine-cytosine (GC) content^14^. An obvious advantage of HRM is that the analysis is performed immediately after the amplification. Thus, post-PCR procedures such as DNA sequencing, which is not a cost-effective method for developing countries, were not required. However, no studies have focused on designing and developing HRM assays to distinguish *Limnonectes* species from each other. In this study, the HRM was therefore developed to identify *Limnonectes* species in Thailand, particularly the two sympatric fanged frogs (*L. bannaensis* and *L. taylori*). This identification method could contribute to future ecological and systematic studies of the target species.

## Results

### Nucleotide analysis

**Figure 2:**
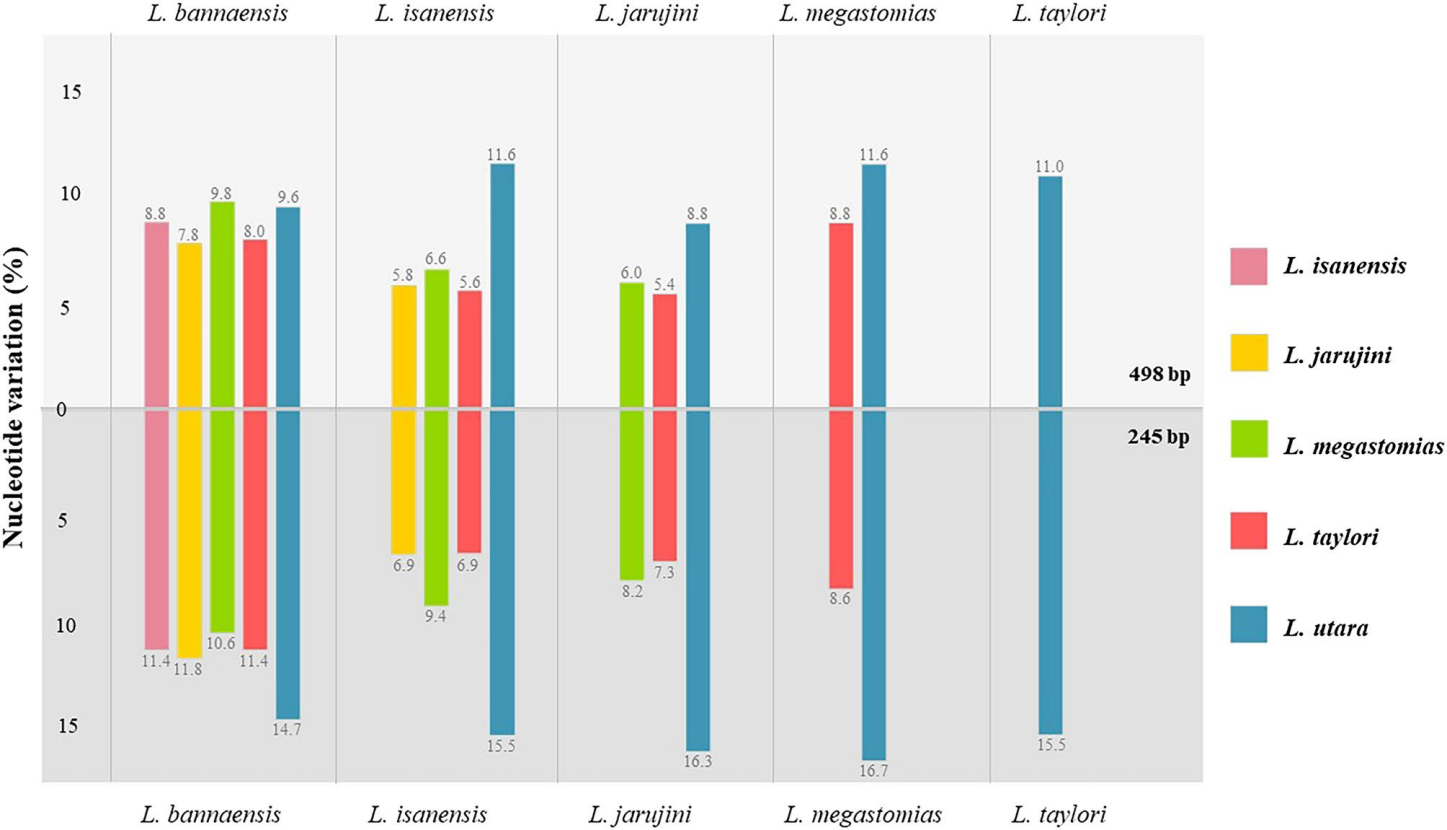
Showing sequence divergence (%) in the mitochondrial 16S rRNA gene between six tested species. Sequence length used in the analyses were 498 bp (upper) and 245 bp (below).

Following detailed morphological comparisons and DNA sequence analyses, we here confirm that twelve *Limnonectes* specimens from Nan province, three from Ratchaburi province, two from Loei Province, and one from Chiang Mai Province were *L. bannaensis*, *L. jarujini*, *L. isanensis*, and *L. taylori*, respectively. Sequence data of the six *Limnonectes* species (*L. bannaensis, L. isanensis, L. jarujini, L. megastomias, L. taylori,* and *L. utara*) were then further analysed for evaluation of HRM success. Nucleotide variation and GC content are shown in Table 1 and Fig. 2. Sequence divergence in the mitochondrial 16S rRNA gene between the six species within the *L. kuhlii* complex was 19.08% (sequence length 498 bp). Nucleotide variation comparing each sequence pair was found to be highest between *L. utara* and *L. megastomias* and between *L. utara* and *L. isanensis,* both at 11.6%. Where the least divergence in their 16S rRNA sequences was between *L. jarujini* and *L. taylori* (5.4%). However, when analysing the amplified fragment generated by HRM16S primers (sequence length 245 bp), nucleotide variation among the six *Limnonectes* species increased to 25.3% (62/245 bp). The highest nucleotide variation in each sequence pair was also between *L. utara* and *L. megastomias* (16.7%). GC content ranges from 38.0 to 46.2%.

**Table 1:** Sequence divergence and GC content in the mitochondrial 16S rRNA gene between the six species within the *L. kuhlii* complex. L = 498 bp fragment, S = 245 bp fragment.

Species	% Nucleotide variation												% GC content	
	*L. bannaensis*		*L. isanensis*		*L. jarujini*		*L. megastomias*		*L. taylori*		*L. utara*			
	L	S	L	S	L	S	L	S	L	S	L	S	L	S
*L. bannaensis*	–	–	8.8	11.4	7.8	11.8	9.8	10.6	8.0	11.4	9.6	14.7	45.9	42.2
*L. isanensis*	–	–	–	–	5.8	6.9	6.6	9.4	5.6	6.9	11.6	15.5	42.0	38.0
*L. jarujini*	–	–	–	–	–	–	6.0	8.2	5.4	7.3	8.8	16.3	43.5	40.5
*L. megastomias*	–	–	–	–	–	–	–	–	8.8	8.6	11.6	16.7	43.0	39.4
*L. taylori*	–	–	–	–	–	–	–	–	–	–	11.0	15.5	43.4	41.1
*L. utara*	–	–	–	–	–	–	–	–	–	–	–	–	46.2	42.7

### Simulation HRM (uMELT Quartz)

**Figure 3:**
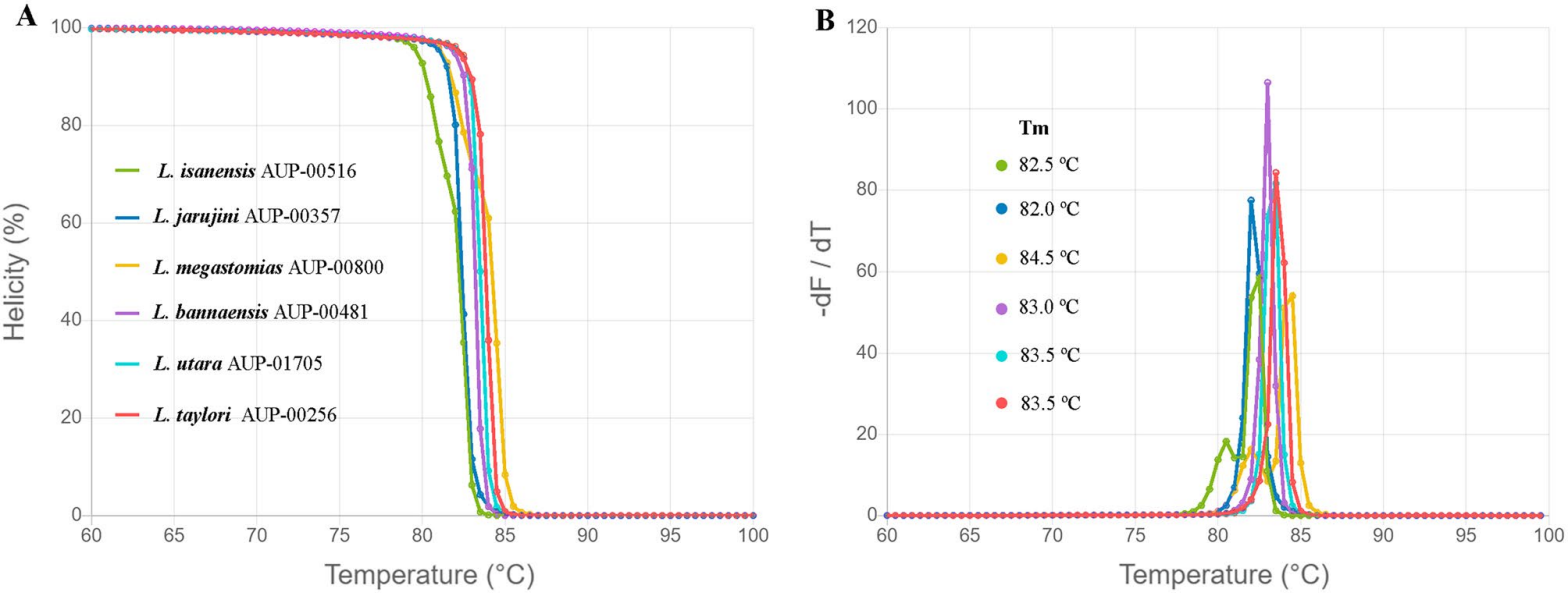
Predicted melting curve of *Limnonectes* species found in Thailand (*L. bannaensis, L. isanensis, L. jarujini, L. megastomias, L. taylori* and *L. utara*) generated from uMELT Quartz. (**A**) normalised melting curves and (**B**) derivative plots showing meting temperature (T_m_) of each sample.

Before performing an in vitro HRM, the DNA sequences were input into a simulation HRM called uMELT Quartz to determine the shape of the melting curve for each *Limnonectes* species. The derivative HRM curves (− dF/dT) and normalised melting curves generated by the programme are shown in Fig. 3A,B. There were differences among the generated melting curves of each species, although the melting plots of *L. bannaensis, L. taylori,* and *L. utara* were highly similar to each other. In addition, as can be seen in Fig. 3B, the melting temperatures (T_m_) of the tested samples range from 82.0 to 84.5 °C. The predicted T_m_ of *L. megastomias* (84.5 °C) was the highest among the six species, while that of *L. jarujini* (82.0 °C) was the lowest.

### In vitro HRM

**Figure 4:**
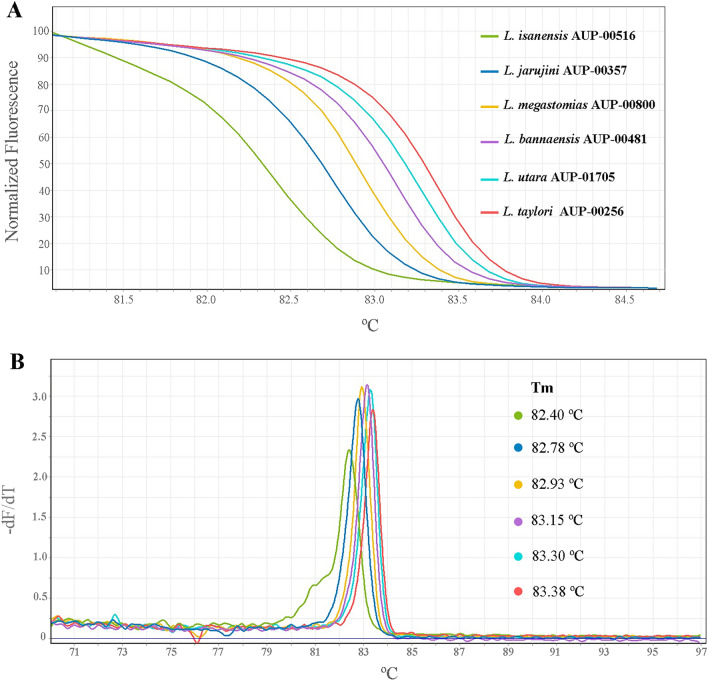
HRM assay of six *Limnonectes* species found in Thailand (*L. bannaensis, L. isanensis, L. jarujini, L. megastomias, L. taylori* and *L. utara*). (**A**) normalised melting curves and (**B**) derivative plots showing meting temperature (T_m_) of each sample.

The HRM assay using the 16S rRNA primer set developed in this study was designed to distinguish the six *Limnonectes* species found in Thailand. The results of the melting curve and T_m_ obtained were consistent with those predicted from uMELT Quartz (Fig. 4A,B) with a slightly different T_m_ value and a more discriminatory difference in the melting curve. All tested *Limnonectes* species were easily differentiated from each other by comparing the differences in the melting curve shapes (Fig. 4A). Melting curves were obtained by plotting the negative derivative of the fluorescence intensity with respect to temperature (− dF/dT) versus temperature (T). The T_m_ value for each species was automatically obtained by identifying the peak of the corresponding melting curve, T_m_ of the tested samples ranged from 82.40 to 83.38 °C.

**Figure 5:**
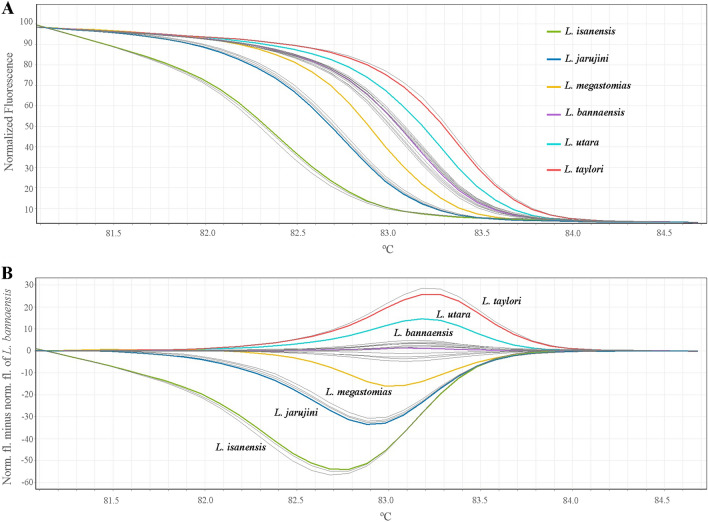
HRM results of 18 analysed *Limnonectes* samples (gray color) with the 16S rRNA primers. (**A**) Normalised curves and (**B**) Difference curves all five *Limnonectes* species comparing with *L. bannaensis*.

The HRM assay was then used to confirm the species of 18 *Limnonectes* samples by comparing them with the reference samples. Similarly, all samples were determined to be the same species based on morphological identification by experts (Table 2). Based on the generating melting curves, from 18 *Limnonectes* samples, 12 were found to be *L. bannaensis*, three were *L. jarujini*, three were *L. isanensis*, and one was *L. taylori* (Figs. 5A,B), with a confidence interval > 90%, ranging from 90.86 to 99.09. Moreover, in our assay, the Sanger sequencing analysis was carried out in parallel for all the field samples, and the results showed that 100% accuracy was achieved from the HRM analysis.

**Table 2:** Species determination via HRM comparing with Morphological and sequences analyses.

ID	Morphological and sequences analyse	HRM analysis		%CI
		Reference	Species	
Lim-1	*L. bannaensis*	AUP-00481	*L. bannaensis*	99.09
Lim-2	*L. bannaensis*	AUP-00481	*L. bannaensis*	98.67
Lim-3	*L. bannaensis*	AUP-00481	*L. bannaensis*	90.86
Lim-4	*L. isanensis*	AUP-00516	*L. isanensis*	98.28
Lim-5	*L. isanensis*	AUP-00516	*L. isanensis*	98.49
Lim-6	*L. bannaensis*	AUP-00481	*L. bannaensis*	92.43
Lim-7	*L. bannaensis*	AUP-00481	*L. bannaensis*	93.93
Lim-8	*L. bannaensis*	AUP-00481	*L. bannaensis*	95.88
Lim-9	*L. jarujini*	AUP-00357	*L. jarujini*	91.62
Lim-10	*L. jarujini*	AUP-00357	*L. jarujini*	98.92
Lim-11	*L. bannaensis*	AUP-00481	*L. bannaensis*	94.33
Lim-12	*L. bannaensis*	AUP-00481	*L. bannaensis*	99.30
Lim-13	*L. bannaensis*	AUP-00481	*L. bannaensis*	94.10
Lim-14	*L. bannaensis*	AUP-00481	*L. bannaensis*	97.25
Lim-15	*L. bannaensis*	AUP-00481	*L. bannaensis*	98.18
Lim-16	*L. bannaensis*	AUP-00481	*L. bannaensis*	94.39
Lim-17	*L. jarujini*	AUP-00357	*L. jarujini*	96.83
Lim-18	*L*. *taylori*	AUP-00256	*L*. *taylori*	92.47

## Discussion

An accurate and rapid identification is essential to enhance knowledge of their ecology and provide the information needed for appropriate conservation planning^15,16^. Identification at the species level using morphological characteristics for certain animal groups, especially amphibians with cryptic species, can be challenging^17^. As mentioned earlier, *Limnonectes* are potentially important for biodiversity assessments, but there now appear to be many more cryptic species than previously estimated. Especially in widespread complexes, such as the *Limnonectes kuhlii* complex, *Limnonectes* displays an extraordinarily high level of morphological similarity^2^. The recent discovery of two *Limnonectes* species in Thailand indicates the sympatric occurrence of *L. taylori* and *L. bannaensis* and highlights the need for a reliable species identification approach other than using morphological characters^5^. The two sympatric species are hardly distinguished from each other based on their morphology. In addition, species identification of tadpoles, juveniles, and adult females of *Limnonectes* is still a difficult task.

An alternative species identification approach that has been widely used is DNA barcoding (use of short DNA sequences for identification). Over the last four decades, partial fragments of mtDNA have been developed to be used as DNA barcodes for identifying animals at the species level. The 16S rRNA mitochondrial gene region is one of the most used for amphibians (e.g., Refs.^18,19^). Although it seems that the advent of recent technologies has made sequencing DNA faster and cheaper, this is not the case in several developing countries. High resolution melting (HRM) analysis has been a promising molecular tool for species identification as it is rapid, simple, cost-effective (requires a fluorescent dye and typical PCR reagents), and a sequencing-free method^20^. HRM analyses samples based on their disassociation behaviour and generates melt curves of product DNA fragments^14^. Samples are discriminated based on their composition, length, and guanine-cytosine (GC) content^21^. In 2019, Everman and Wang^9^ utilised the HRM of COI identifiers (COI-HRM) to differentiate anurans. Their findings indicated that HRM is a straightforward and efficient method for distinguishing morphologically similar animal species. Remarkably, the method has not been applied to the *Limnonectes kuhlii* complex, which consists of species with extremely similar morphology. Here, HRM assays using 16S rRNA primers were designed and developed to distinguish six *Limnonectes* species found in Thailand.

In our case, the DNA sequencing was carried out in parallel for the field samples, and thus we can perform nucleotide analysis (finding nucleotide variation and GC content, the two important factors that contribute to the success of HRM) and use them in simulation HRM (uMELT Quartz) to evaluate the success potential of the developed HRM analysis. Sequence variations among the tested *Limnonectes* species were as high as 25.3%, which was a good indicator for success in HRM analysis. Although the least variation between two species was found to be as small as 6.9% (*L. jarujini*–*L. isanensis* and *L. jarujini*–*L. taylori*), HRM could still be used to differentiate between them. As not only the variation of the sequences could influence DNA duplex stability but also nucleotide composition. The nearest-neighbour (NN) model shows that the stability of DNA depends on the type and orientation of neighbouring base pairs^22^ and thus the NN effect has been considered in DNA melting analysis works and simulation HRM tools that are used to predict melting curves for DNA duplexes of interest, including the uMELT Quartz^23^.

Several HRM studies have shown that the prediction programme was good enough to indicate whether the in vitro HRM of the interested samples would be successful (e.g., Refs.^24–26^). This was also the case here, as the uMELT Quartz showed that the melting curves of all tested *Limnonectes* species were not identical and clearly separated. The results of the in vitro HRM were comparable to those predicted, with a slightly different T_m_ value, and the melting curves were more different among the six species. This is likely due to the narrower temperature increments (0.1 °C) of in vitro HRM analysis. The narrowest temperature increments that can be set in uMELT Quartz are only 0.5 °C^23^. Thus, better resolution was achieved with the in vitro HRM assays. The HRM was found to be useful when morphological-based identification is difficult, and therefore the method will also contribute to future ecological and systematic studies on the target species.

## Methods

### Ethics statement

All procedures were conducted in accordance with the current laws in Thailand on experimental animals and were approved by the safety management committee for experiments of the Institutional Ethical Committee of Animal Experimentation of the University of Phayao, Phayao, Thailand (project number 640204004). The study also followed the recommendations in the ARRIVE guidelines.

### Samples

Field surveys were conducted in Chiang Mai, Loei, Nan, Sa Kaeo, and Ratchaburi provinces, Thailand, in December 2017. Eighteen individuals of *Limnonectes* from a hitherto unknown population were collected (Table 3). Tissue samples of the liver from all specimens were preserved in 95% ethanol for further analysis. Specimens were then fixed with 10% formalin for 24 h and subsequently transferred to 70% ethanol. All fixed specimens were deposited at the University of Phayao. Six specimens with 16S rRNA DNA sequences deposited in GenBank were used as reference samples in HRM analysis (Table 3). All samples were examined and identified by experts in the previous study^5^.

**Table 3:** Details of *Limnonectes* specimens used in HRM assay. *Indicates reference samples in the HRM analysis with their sequence accession numbers.

ID	Species	Locality	Accession number	Reference
AUP-00481*	*Limnonectes bannaensis*	Bo klue District, Nan Province	MZ493348	Suwannapoom et al.^5^
AUP-00516*	*Limnonectes isanensis*	Phu Luang District, Loei Province	OR272275	This study
AUP-00357*	*Limnonectes jarujini*	Suan Pueng District, Ratchaburi Province	OR272276	This study
AUP-00800*	*Limnonectes megastomias*	Muang Sa Kaeo, Sa Kaeo Province	OR272278	This study
AUP-00256*	*Limnonectes taylori*	Doi Saket District, Chiang Mai Province	OR272277	This study
AUP-01705*	*Limnonectes utara*	Bannang Sata District, Yala Province	MZ493344	Suwannapoom et al.^5^
Lim-1	*Limnonectes* sp.	Pua District, Nan Province	–	–
Lim-2	*Limnonectes* sp.	Pua District, Nan Province	–	–
Lim-3	*Limnonectes* sp.	Pua District, Nan Province	–	–
Lim-4	*Limnonectes* sp.	Phu Luang District, Loei Province	–	–
Lim-5	*Limnonectes* sp.	Phu Luang District, Loei Province	–	–
Lim-6	*Limnonectes* sp.	Pua District, Nan Province	–	–
Lim-7	*Limnonectes* sp.	Pua District, Nan Province	–	–
Lim-8	*Limnonectes* sp.	Pua District, Nan Province	–	–
Lim-9	*Limnonectes* sp.	Suan Pueng District, Ratchaburi Province	–	–
Lim-10	*Limnonectes* sp.	Suan Pueng District, Ratchaburi Province	–	–
Lim-11	*Limnonectes* sp.	Pua District, Nan Province	–	–
Lim-12	*Limnonectes* sp.	Pua District, Nan Province	–	–
Lim-13	*Limnonectes* sp.	Pua District, Nan Province	–	–
Lim-14	*Limnonectes* sp.	Pua District, Nan Province	–	–
Lim-15	*Limnonectes* sp.	Pua District, Nan Province	–	–
Lim-16	*Limnonectes* sp.	Pua District, Nan Province	–	–
Lim-17	*Limnonectes* sp.	Suan Pueng District, Ratchaburi Province	–	–
Lim-18	*Limnonectes* sp.	Chom Thong District, Chiang Mai Province	–	–

### Morphological examination, DNA extraction, and sequencing

All specimens were morphologically examined by experts accordingly Suwannapoom et al.^5^ Genomic DNA and Sanger sequencing of all 18 specimens (Lim1–Lim18) were then carried out following Suwannapoom et al.^5^ Briefly, DNA was extracted from liver tissues using a standard phenol–chloroform extraction protocol of Sambrook et al.^27^ A partial fragment of the mitochondrial 16S rRNA was amplified by polymerase chain reaction (PCR) using the following primers: 16SAR (5′-CGCCTGTTTAYCAAAAACAT-3′) and 16SBR (5′-CCGGTYTGAACTCAGATCAYGT-3′; Kocher et al., 1989). PCR amplifications were performed in a 25 µL reaction volume with the following cycling conditions: an initial denaturing step at 95 °C for 4 min, 35 cycles of denaturing at 94 °C for 40 s, annealing at 55 °C for 16S rRNA for 30 s, extending at 72 °C for 1 min, and a final extension at 72 °C for 10 min. PCR products were sequenced by an ABI 3730xl DNA automated sequencer with both forward and reverse primers.

### Sequence analysis

Two sequence datasets (L and S) with the same number of species but different sequence lengths were constructed for sequence profile analysis. The sequences of the 16S rRNA region of six *Limnonectes* species (*L. bannaensis*, *L. isanensis*, *L. jarujini*, *L. megastomias, L. taylori,* and *L. utara*) generated in previous study^5^ were included in the datasets (Table 3). All sequences in an L dataset contained 498 nucleotides, whereas all sequences in an S dataset were 5′ and 3′ trimmed, which are 245 nucleotides long. Multiple alignments of the sequences in both datasets were performed using MEGA 11 programme^28^ and GC content and nucleotide variation between species (%) were recorded.

### Simulation HRM

Sequences in the S dataset were used here to evaluate the success potential of an HRM assay by determining the melting profile for each *Limnonectes* species. Simulated HRM analyses were performed using uMELT Quartz (melting prediction software) following the user guide^23^. The melting curves of the 16S rRNA regions of the tested species were compared for their efficiency in species discrimination.

### In vitro HRM

HRM analyses were performed according to Osathanunkul et al.^6^ Briefly, the extracted DNA was amplified using the Rotor-Gene Q 5plex HRM system (Qiagen, Hilden, Germany). The reaction mixture for the HRM analysis consisted of a total volume of 20 µL, containing 4 µL of Evagreen HRM Master Mix, 0.4 µL of 10 mM forward primer, 0.4 µL of 10 mM reverse primer, 1 µL of DNA, and 14.2 µL of ddH_2_O. The reaction conditions were as follows: an initial denaturing step at 95 °C for 5 min, followed by 40 cycles of 95 °C for 30 s, 57 °C for 30 s and 72 °C for 20 s. After the last extension step, melting curves were generated in which temperatures increased from 70 to 95 °C at 0.1 °C/s. The nucleotides of HRM16S forward primer are 5′-CGAGAAGACCCTATGGAGCTT-3′ and the reverse primer are 5′-AATGGATTGCGCTGTTATCCC-3′. Melting curves were generated after the last extension step at a temperature range of 65–95 °C, rising by 0.1 °C every 2 s. The fluorescence acquisition setting recommended by the manufacturer. The melting profiles were normalised by adjusting before and after the major fluorescence decrease, respectively. The melting data of the samples were recorded and analysed using Rotor-gene Q series software v.2.3.1. To identify species of the 18 tested samples, normalised melting plot of reference samples (Table 3) were generated and used to compare with the curves of tested samples. The percentage of confidence interval (CI) of ≥ 90% was used to determine whether the melting plot of sample showed similarity with the reference’s curve. HRM analyses were carried out in triplicate.

## Data Availability

GenBank Accession Numbers of datasets generated and/or analysed during the current study are provided in Table 3.

## References

[1] Bickford D (2007). Cryptic species as a window on diversity and conservation. Trends Ecol. Evol..

[2] McLeod DS (2010). Of least concern? Systematics of a cryptic species complex, *Limnonectes kuhlii* (Amphibia; Anura, Dicroglossidae). Mol. Phylogenet. Evol..

[3] Frost, D. R. Amphibian Species of the World: an Online Reference. Version 6.1. Electronic Database. American Museum of Natural History, New York, USA. http://research.amnh.org/herpetology/amphibia/index.html (accessed 28 Aug 2022) (2022).

[4] Matsui M, Panha S, Khonsue W, Kuraishi N (2010). Two new species of the *kuhlii* complex of the genus *Limnonectes* from Thailand (Anura, Dicroglossidae). Zootaxa.

[5] Suwannapoom C (2021). First records of the fanged frogs Limnonectes bannaensis Ye, Fei & Jiang, 2007 and *L. utara* Matsui, Belabut & Ahmad, 2014 (Amphibia: Anura: Dicroglossidae) in Thailand. Biodivers. Data J..

[6] Osathanunkul M, Madesis P, Boer HD (2015). Bar-HRM for authentication of plant-based medicines: evaluation of three medicinal products derived from Acanthaceae species. PLoS One.

[7] Osathanunkul M, Suwannapoom C, Osathanunkul K, Madesis P, De Boer H (2016). Evaluation of DNA barcoding coupled high resolution melting for discrimination of closely related species in phytopharmaceuticals. Phytomedicine.

[8] Song M, Li J, Xiong C, Liu H, Liang J (2016). Applying high-resolution melting (HRM) technology to identify five commonly used Artemisia species. Sci. Rep..

[9] Everman S, Wang SY (2019). Distinguishing Anuran species by high-resolution melting analysis of the COI barcode (COI-HRM). Ecol. Evol..

[10] Baudrin G, Roy V, Gigon A, Dupont L (2020). Bar-HRM for identification of cryptic earthworm species. Pedobiologia.

[11] Chen C (2021). High-resolution melting analysis of COI sequences distinguishes Pufferfish Species (Takifugu spp.) in China. J. Agric. Food Chem..

[12] Miller M, Zorn J, Brielmeier M (2015). High-resolution melting curve analysis for identification of Pasteurellaceae species in experimental animal facilities. PLoS One.

[13] Pakbin B (2022). Development of high-resolution melting (HRM) assay to differentiate the species of Shigella isolates from stool and food samples. Sci. Rep..

[14] Wittwer CT, Reed GH, Gundry CN, Vandersteen JG, Pryor RJ (2003). High-resolution genotyping by amplicon melting analysis using LCGreen. Clin. Chem..

[15] Carvalho-Batista A, Terossi M, Zara FJ, Mantelatto FL, Rogerio C (2019). Costa A multigene and morphological analysis expands the diversity of the seabod shrimp Xiphopenaeus Smith, 1869 (Decapoda: Penaeidae), with descriptions of two new species. Sci. Rep..

[16] França NFC (2019). Farfantepenaeus subtilis (Pérez-Farfante, 1967) and *F. brasiliensis* (Latreille, 1817) (Decapoda, Penaeidae): Ontogenetic comparison using the combined analysis of secondary sexual characters and molecular markers. Fish. Res..

[17] Stuart BL, Inger RF, Voris HK (2006). High level of cryptic species diversity revealed by sympatric lineages of Southeast Asian Forest frogs. Biol. Lett..

[18] Vences M, Thomas M, van der Meijden A, Chiari Y, Vieites DR (2005). Comparative performance of the 16S rRNA gene in DNA barcoding of amphibians. Front. Zool..

[19] Chan KO, Hertwig ST, Neokleous DN, Flury JM, Brown RM (2022). Widely used, short 16S rRNA mitochondrial gene fragments yield poor and erratic results in phylogenetic estimation and species delimitation of amphibians. BMC Ecol. Evol..

[20] Vossen RH, Aten E, Roos A, den Dunnen JT (2009). High-Resolution Melting Analysis (HRMA) more than just sequence variant screening. Hum. Mutat..

[21] Garritano S (2009). Determining the effectiveness of High Resolution Melting analysis for SNP genotyping and mutation scanning at the TP53 locus. BMC Genet..

[22] Turner DH, Mathews DH (2010). NNDB: The nearest neighbor parameter database for predicting stability of nucleic acid secondary structure. Nucleic Acids Res..

[23] Dwight Z, Palais R, Wittwer CT (2011). uMELT: Prediction of high-resolution melting curves and dynamic melting profiles of PCR products in a rich web application. Bioinformatics..

[24] Liu B (2013). Development of InDel markers for *Brassica rapa* based on whole-genome re-sequencing. Theor. Appl. Genet..

[25] Osathanunkul M, Sawongta N, Madesis P, Pheera W (2022). Bar-HRM for species confirmation of native plants used in forest restoration in Northern Thailand. Forests.

[26] Baoutina A, Bhat S (2022). Novel design of nucleic acid standards for hydrolysis probe-based PCR with melting analysis. Gene Ther..

[27] Sambrook, J., Fritsch, E. F. & Maniatis, T. *Molecular Cloning. A Laboratory Manual*, 2nd ed. (Cold Spring Harbor Laboratory Press, 1989).

[28] Tamura K, Stecher G, Kumar S (2021). MEGA11: Molecular evolutionary genetics analysis version 11. Mol. Biol. Evol..

